# The therapeutic potential of targeting the PI3K pathway in pediatric brain tumors

**DOI:** 10.18632/oncotarget.13781

**Published:** 2016-12-02

**Authors:** Hazel A. Rogers, Jasper Estranero, Keshni Gudka, Richard G. Grundy

**Affiliations:** ^1^ Childrens Brain Tumour Research Centre, School of Medicine, University of Nottingham, Nottingham, UK

**Keywords:** PI3K pathway, brain tumor, pediatric, therapy, cancer

## Abstract

Central nervous system tumors are the most common cancer type in children and the leading cause of cancer related deaths. There is therefore a need to develop novel treatments. Large scale profiling studies have begun to identify alterations that could be targeted therapeutically, including the phosphoinositide 3-kinase (PI3K) signaling pathway, which is one of the most commonly activated pathways in cancer with many inhibitors under clinical development. PI3K signaling has been shown to be aberrantly activated in many pediatric CNS neoplasms. Pre-clinical analysis supports a role for PI3K signaling in the control of tumor growth, survival and migration as well as enhancing the cytotoxic effects of current treatments. Based on this evidence agents targeting PI3K signaling have begun to be tested in clinical trials of pediatric cancer patients. Overall, targeting the PI3K pathway presents as a promising strategy for the treatment of pediatric CNS tumors. In this review we examine the genetic alterations found in the PI3K pathway in pediatric CNS tumors and the pathological role it plays, as well as summarizing the current pre-clinical and clinical data supporting the use of PI3K pathway inhibitors for the treatment of these tumors.

## INTRODUCTION

Neoplasms of the CNS are the most common type of solid tumor that occur in children and the leading cause of cancer related deaths [1]. Currently prognosis for more aggressive types is relatively poor [2–4] and there is a need to identify novel therapies. Many pediatric CNS tumors have undergone extensive genomic and molecular characterization enabling identification of genetic and epigenetic alterations which could be targets for novel therapies [5–9]. One such target is the phosphoinositide 3-kinase (PI3K) pathway.

## PI3K PATHWAY

The PI3K pathway is one of the most commonly activated pathways in cancer. PI3Ks are lipid kinases that activate a signaling cascade which controls diverse biological functions including cellular proliferation, survival and motility. PI3Ks can be broadly divided into three structural classes; Class I, II and III.

Class I PI3Ks can be further divided into two subtypes, Class IA and IB, dependent on their method of activation. Class IA PI3Ks are activated by receptor tyrosine kinases (RTKs), G-protein coupled receptors (GPCRs) and oncogenes, whereas Class IB PI3Ks are activated by GPRCs only [10]. Class IA PI3Ks are composed of a p110 catalytic subunit and a p85 regulatory subunit. There are three isoforms of the p110 catalytic subunit; p110α, p110β, p110δ and three isoforms of the p85 regulatory subunit; p85α, p85β, p55γ. Class IB PI3Ks consist of a p110γ catalytic subunit in complex with either p101 or p87 regulatory subunits. Signaling through class I PI3Ks regulates cell growth and metabolism [11].

**Figure 1:**
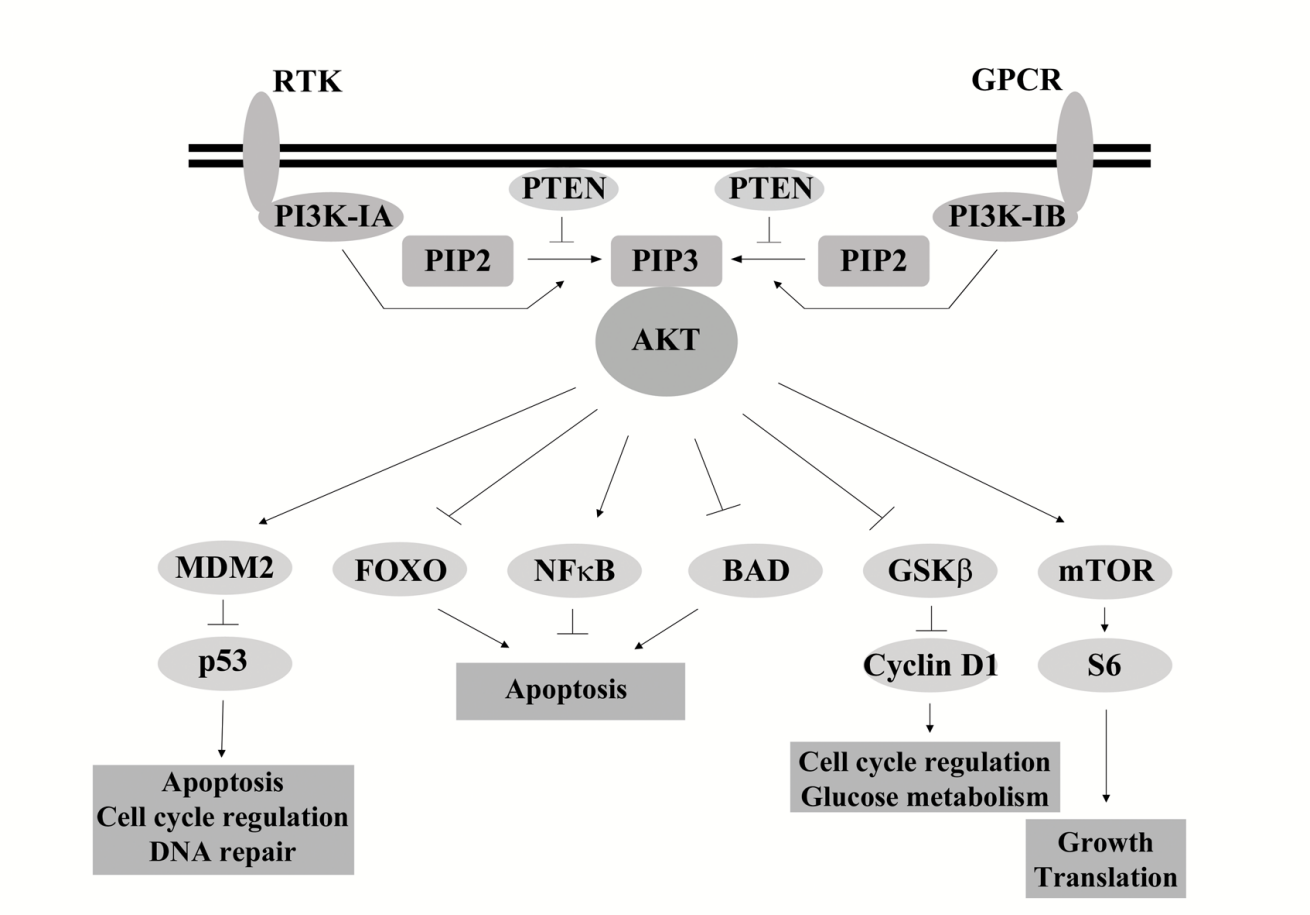
Overview of Class I PI3K signaling Following activation of receptors, through ligands such as growth factors or cytokines, PI3K is recruited to the membrane where the regulatory subunit interacts with the receptor. The activated catalytic subunit converts PIP2 to PIP3. PTEN negatively regulates this reaction, converting PIP3 back to PIP2. PIP3 recruits AKT to the membrane where it is activated through phosphorylation. Once activated AKT regulates a range of targets, of which a small set of examples are presented, activating or inhibiting their action through phosphorylation.

Class I PI3Ks activate canonical PI3K/AKT signaling. When ligands, such as growth factors or cytokines, bind to their receptor PI3K is recruited to the membrane where the regulatory subunit directly interacts with the activated receptor. After activation, class I PI3K phosphorylates the lipid phosphatidylinositol-4,5-bisphosphate (PIP_2_) to produce phosphatidylinositol-3,4,5-bisphosphate (PIP_3_). This reaction is negatively regulated by phosphate and tensin homolog (PTEN) which reduces levels of PIP_3_ by converting it back to PIP_2_. PIP_3_ forms a docking site for the recruitment of many proteins to the plasma membrane including the serine threonine kinase V-Akt murine thymoma viral oncogene homolog (AKT), where it is activated by phosphorylation by phosphoinositide-dependent protein kinase 1 (PDK1) and mammalian target of rapamycin complex 2 (mTORC2). Once activated, AKT regulates key cellular activities downstream, including glycogen synthesis by the forkhead family of transcription factors (FOXOs) and apoptosis through p53, BAD and NfκB (Figure 1) [11].

Alternative mechanisms of PI3K pathway activation can be mediated by small GTPases such as Ras. Ras is capable of activating Class I PI3K isoforms p110α, p110γ and p110δ by binding to the RAS-Binding Domain. Class I p110β can also be regulated by the Rho family of GTPases, particularly RAC1 and CDC42 [12].

One target of PI3K signaling that has commonly been implicated in cancer is mammalian target of rapamycin (mTOR). Signaling through mTOR regulates key cellular activities including cell growth and protein synthesis [13]. mTOR forms two complexes to exert its downstream actions, mammalian target of rapamycin complex 1 (mTORC1) and mammalian target of rapamycin complex 2 (mTORC2), which are differentially regulated by upstream signals. Both can be regulated by PI3K signaling. AKT phosphorylates tuberous sclerosis 2 (TSC2) and proline rich AKT substrate 40 kDa (PRAS40) which attenuates their inhibitory effects on mTORC1 [14]. PI3K signaling has recently been linked to activation of mTORC2 where PIP_3_ has been identified as a direct upstream activator [15].

Class II PI3Ks consist of a single catalytic subunit of which there are three isoforms PI3KC2α, PI3KC2β and PI3KC2γ. They can be activated by RTKs, cytokine receptors and integrins. Their specific cellular functions remain poorly understood. Recent evidence suggests signaling may play a role in angiogenesis, growth and survival [16]. Class III PI3K consists of a single catalytic subunit VPS34. Signaling through this PI3K class has been linked to roles in cellular trafficking and regulation of autophagy [17].

## PI3K SIGNALING AND CANCER

Signaling through class I PI3Ks has commonly been implicated in cancer. A number of genetic alterations resulting in pathway activation have been identified. PTEN is one of the most frequently inactivated tumor suppressor genes, with loss commonly occurring through deletions and inactivating mutations [18]. Loss of PTEN results in a lack of control of PIP_3_ levels and un-restrained PI3K signaling.

PI3KCA, which encodes the p110α catalytic subunit of class IA PI3K, is frequently mutated in some of the most common human cancers [19, 20]. 80% of reported mutations cause amino acid substitutions in the helical and kinase domains and result in activation of class IA PI3K [21]. Mutations can also occur in p110β and p110δ but are less common. Alternatively amplification or overexpression of these genes has been reported [22]. Mutations have also been identified in the regulatory PI3K subunits, usually in the form of substitutions or in-frame deletions or insertions. This includes PI3KR1 which encodes the regulatory subunit p85α, where mutations have been found in 10% of glioblastomas [19].

Activating mutations or amplifications of AKT1/2 have also been identified [23]. Mutations are rarely seen in PDPK1 but amplification or overexpression has been detected in breast cancer [24]. Class I PI3K signaling activation can also result from activating mutations in RAS, the most highly mutated oncogene in cancer [25].

Class II PI3K isoforms are less well understood. However, they have been implicated in cancer. Amplification and somatic mutations have been identified in PIK3C2B [26]. However, the functional implications of these alterations are not understood.

Mutations have also been identified in the negative regulators of mTOR signaling TSC1 and TSC2. Mutations in these genes occur in the familial syndrome tuberous sclerosis complex (TSC), a rare autosomal dominant disorder. Patients with TSC develop benign tumors in multiple organs including brain, heart, kidney, lungs and skin [27]. Loss of TSC1/2 also occurs at low frequency in sporadic cancer [28, 29].

**Figure 2:**
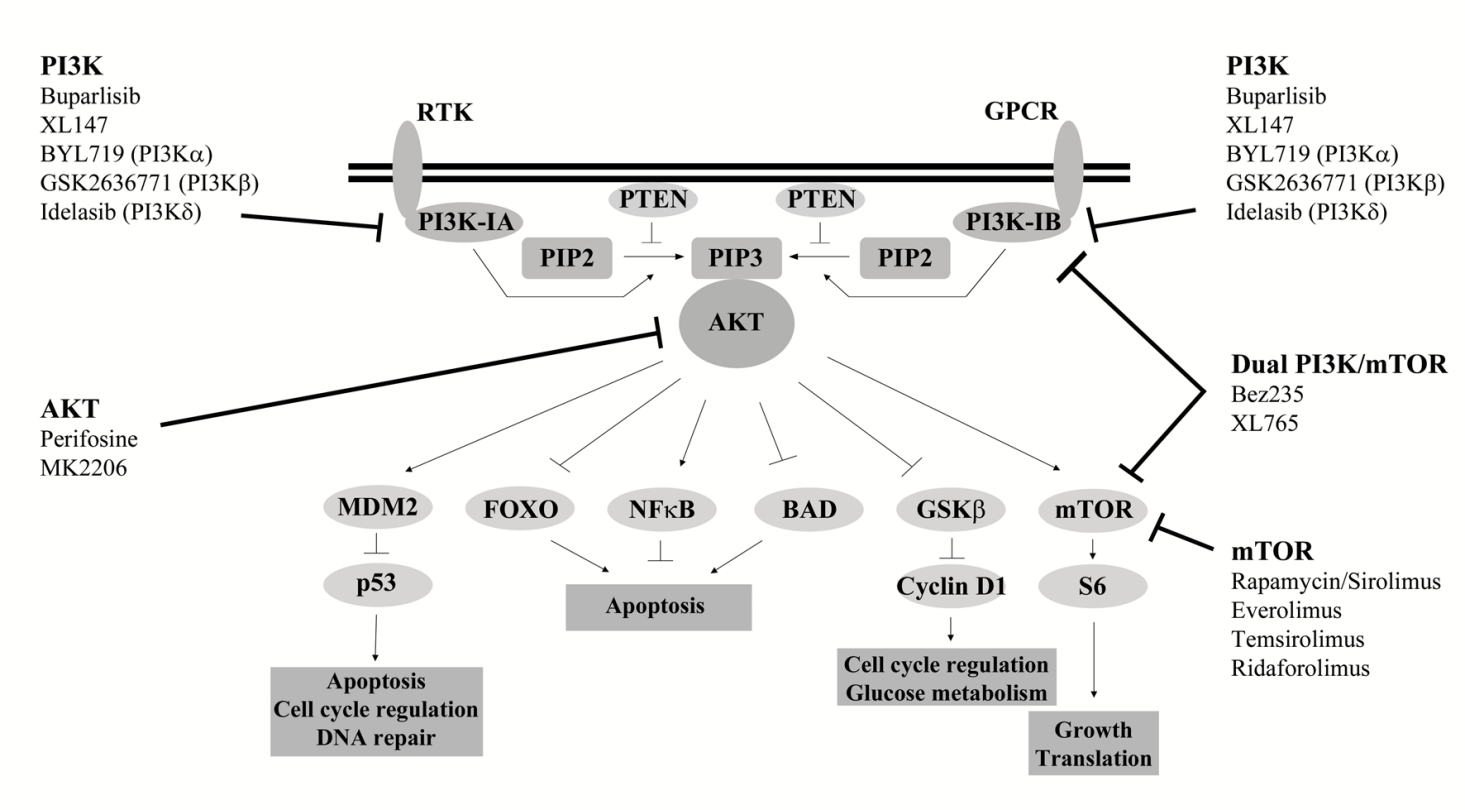
PI3K pathway inhibitors in clinical development Examples of inhibitors in clinical development which target various nodes of the PI3K signalling pathway, including class I PI3K (pan or isoform), AKT and mTOR. Specific targets are given in brackets.

The common occurrence of PI3K pathway deregulation in cancer has sparked interest in the development of inhibitors targeting PI3K signaling, with over 30 inhibitors being trialed clinically (Figure 2). The first generation of PI3K inhibitors, wortmanin and LY294002, are broad spectrum with their clinical effectiveness limited by high toxicity and poor solubility [11]. Currently, three main classes of PI3K inhibitors are in clinical development; pan-Class I PI3K, dual pan-Class I PI3K/mTOR and PI3K isoform specific [30]. The first inhibitor to be approved for treatment was the PI3Kδ isoform inhibitor idelalisib for treatment of B-cell malignancies [31]. However, in general only modest effects have been seen in trials of PI3K pathway inhibitors to date [30]. Inhibitors targeting AKT have also been developed. The most advanced clinically is perifosine, a lipid based phosphatidylinositol targeting the homology domain of AKT and impeding its translocation to the plasma membrane [32].

Agents targeting mTOR signaling are more clinically advanced than other nodes of the pathway. The first inhibitor to be discovered was rapamycin, a bacterially derived product which was originally developed as a fungicidal and immunosuppressive drug [11, 33]. Analogues of rapamycin with better pharmacological properties, called rapalogues, such as everolimus and temsirolimus have subsequently been developed [34]. Improved survival has been seen with rapalogues in patients with some cancers [33] and two rapalogues, temsirolimus and everolimus, are currently approved for cancer treatment [35–37]. However, modest outcomes have been seen in clinical trials for other tumor types [33]. Additionally, rapalogues only inhibit mTORC1. Inhibitors that target both mTORC1 and 2 have begun to be developed which induce more potent inhibition of mTORC1 and also block mTORC2 [38].

Recent evidence also suggests that PI3K inhibition can be used not only to target tumor cells directly but also the surrounding stroma, including tumor vasculature and infiltrating immune cells [39, 40]. The clinical efficacy of idelalisib in the treatment of B cell malignancies is in part due to interference in the interaction of cancer cells with the surrounding tumor stroma [41]. Studies also suggest PI3K inhibition could be used to promote a tumor specific immune response [42].

## PI3K PATHWAY IN PEDIATRIC BRAIN TUMORS

### Pediatric high grade glioma (pHGG)

pHGG account for 5-10% of all pediatric CNS tumors. Prognosis for children with pHGG is dismal with a 5 year survival rate of less than 20% [2]. pHGG encompass a number of variants including glioblastoma, anaplastic astrocytoma, anaplastic oligodendroglioma, gliomatosis cerebri and diffuse intrinsic pontine glioma (DIPG).

Activation of PI3K signaling has been found in a high percentage of pHGG, which in some studies was linked to a poorer prognosis [5, 43, 44]. This is similar to adult HGGs where PI3K pathway activation has also commonly been seen and found to be strongly associated with a poorer prognosis [45].

However, the mechanism of aberrant pathway activation differs between adult and pediatric tumors. In adult tumors inactivating PTEN mutations are commonly seen. In contrast, in pediatric cases they are infrequent [46]. However, inactivation of PTEN through promoter methylation has been reported [47], while allelic loss through deletions have been found in DIPG [48]. Activating mutations in PIK3CA have also been detected in pHGG. Gallia et al identified mutations in 21% of pediatric glioblastomas which was similar to rates identified in adult glioblastomas (17%) [49]. Grill et al also identified PIK3CA mutations in 15% of DIPGs [50].

Pathway activation through amplification or overexpression of growth factor receptors is also common. Amplification or overexpression of EGFR was commonly found in adult HGGs but was rarely seen in pHGG [8]. However, overexpression has been reported [44, 46]. EGFRvIII mutations have also been identified [51, 52]. The predominant target of amplification in pHGGs is PDGFRA, particularly in DIPG [8, 48, 51]. Amplifications were found in 11% of non-brainstem pHGGs and 26% of DIPGs. Somatic point mutations in PDGFRA have also been identified in 14% of non-brainstem pHGGs and 5% of DIPGs [53]. Amplification of other members of the PI3K signaling pathway have also been reported at low frequencies [8].

A recent whole genome sequencing study identified frequent activation of PI3K signaling through mutations in 67% of non-brainstem HGGs and 69% of DIPGs, including fusions involving the kinase domain of neurotrophin receptor (NTRK) genes, highlighting the importance of this signaling pathway in these tumors [52]. However, a multiregional sequencing study of DIPG suggested PI3K signaling mutations were sub-clonal rather than driver mutations [54].

### Pediatric low grade glioma (pLGG)

pLGG are the most common type of pediatric brain tumor and account for approximately 40% of cases [55]. pHGG encompass a range of variants with the most common being pilocytic astrocytoma [55]. Prognosis is relatively good, however those that cannot be surgically removed can cause morbidity and there is therefore a need to identify novel therapies [55, 56].

PI3K signaling has been implicated in pLGG. mTOR activation has been associated with subependymal giant cell astrocytoma (SEGA) which occurs in up to 15% of TSC patients [57]. Evidence of PI3K activity has also been found in other types of pLGG. Proteomic analysis demonstrated mTOR activation across pLGG subtypes [58, 59]. mTOR activation has also been linked to clinically aggressive and anaplastic subsets of pilocytic astrocytomas [60]. Additionally, FGFR1 duplications and MYB rearrangements have been shown to activate PI3K signaling in pLGG [56].

Pre-clinical analysis has produced evidence supporting the potential for therapeutic targeting using mTOR inhibitors in pLGG. Decreased growth was seen when two pLGG cell lines were treated with an mTORC1 inhibitor [58]. Kaul et al demonstrated a link between the BRAF fusion commonly found in pLGG and mTOR signaling, where neural stem cell proliferation driven by the BRAF fusion was shown to be mediated through mTOR signaling [61]. This was supported by the significant survival advantage of pLGG flank xenografts treated with the mTOR inhibitor everolimus and BRAF inhibitor PLX4720 compared to each agent alone [62].

### Ependymoma

Ependymoma is the second most common malignant pediatric CNS tumor, accounting for approximately 10% of brain tumors [63]. Prognosis is relatively poor particularly at recurrence, which is common [4]. 90% of pediatric cases are intracranial, 70% occurring in the posterior fossa.

An immunohistochemical analysis of phosphorylated AKT demonstrated positive staining, and inferred pathway activation, in a high percentage of ependymomas (72% of primary tumors), which was significantly linked to a poorer progression free survival. Cyclin D1 was significantly associated with active PI3K signaling suggesting control of cell proliferation [64]. Active PI3K signaling has also been demonstrated in ependymoma cell lines [65].

A definitive mechanism for activation of PI3K signaling has not been determined for ependymoma. Sequencing studies have not identified PIK3CA mutations [7, 64]. Copy number loss of the PTEN loci has been reported [66], however no loss of PTEN protein expression was detected across a large ependymoma cohort [64]. Overexpression of ERBB receptors has been reported at variable frequencies [67, 68]. ERBB2/4 co-expression has also been reported at high frequencies with high co-expression significantly related to tumor proliferation and disease risk [67]. A recent study also demonstrated that the putative ependymoma oncogene AKT2 could drive formation of ependymomas in mice [69]. However, gain of AKT2 has only been detected in approximately 4% of ependymomas [66].

A decrease in cell viability has been demonstrated in ependymoma cell lines following treatment with PI3K pathway inhibitors suggesting the potential for therapeutic targeting [64, 65]. A high throughput drug screen using an ependymoma mouse model also identified inhibitors of IGF, signaling through PI3K, as a significant target [70].

### Medulloblastoma

Medulloblastoma is an embryonal tumor and is the most common malignant brain tumor in children [6]. Recent large scale analysis has enabled researchers to divide medulloblastomas into four molecular subgroups; WNT, SHH, Group 3 and Group 4, which correlate with demographic and clinical factors [6]. Prognosis differs significantly between these groups with the best outcome occurring for patients with WNT subgroup tumors and the poorest for those with a Group 3 tumor [6].

Active PI3K signaling has been detected in a high percentage of medulloblastomas [71–73], and has been shown to be de-regulated by a number of mechanisms. Various growth factor receptors, which can activate PI3K signaling, have been shown to be over-expressed by medulloblastoma cells, through mechanisms including copy number gain and amplification [74–76]. Decreased levels of the tumor suppressor PTEN have frequently been seen, caused by mutations, copy number loss or promoter methylation [73, 77]. PIK3CA mutations have also been identified at a low frequency [78]. PIK3CA overexpression was commonly seen and was associated with active PI3K signaling [72]. PIK3C2B amplifications have been detected [79] as well as overexpression of PI3KC2β and PIK3CG [80]. Amplification of AKT3 has also been identified [81]. Additionally, a recent study demonstrated that Myc induced PI3K signaling drove a metastatic phenotype in orthotopic models [82].

Inhibition of PI3K signaling has demonstrated a role for the pathway in control of medulloblastoma cell growth and migration. Treatment with inhibitors at various nodes of the signaling pathway, including PI3K, AKT and mTOR, resulted in impaired growth and migration of medulloblastoma cells using in vitro and in vivo models [83, 84]. Treatment with pathway inhibitors was also shown to enhance the cytotoxic effects of chemotherapy drugs such as cisplatin and etoposide [71]. Targeting PI3K isoforms specifically has resulted in variable success. Targeting the Class IA isoform p110α or the Class IB isoform p110γ resulted in decreased medulloblastoma cell proliferation and enhanced effects of chemotherapy [72, 84, 85]. However, targeting of other Class IA isoforms was not so effective [72]. Reduced proliferation was also seen following inhibition of the Class II isoform PIK3C2β [86].

PI3K signaling has also been linked to tumor resistance in medulloblastoma. Inhibition of PI3K signaling in a sonic hedgehog pathway induced medulloblastoma mouse model delayed the development of smo-induced resistance [87].

### Atypical teratoid/rhabdoid tumor (ATRT)

ATRT is a highly malignant embryonal tumor, distinguished by alterations in the tumor suppressor gene INI1 [3]. It is currently considered one of the most malignant and difficult pediatric tumors to cure [88]. Patients respond poorly to chemotherapy and radiotherapy and prognosis is poor [3].

Studies have implicated PI3K signaling in the control of ATRT cell growth. ATRT cell lines were found to overexpress RTKs, including insulin receptor and insulin-like growth factor-I receptor, as well as PI3K signaling components, compared to normal brain. PI3K signaling was also shown to play a crucial role in ATRT cell proliferation induced by insulin signaling with evidence that induced signaling was mediated specifically through the Class IA PI3K isoform p110α [89]. Pharmacological inhibitors of PI3K signaling have also been shown to impair the growth of ATRT cells [88].

### Embryonal tumors

Embryonal tumors previously classified as central nervous system primitive neuroectodermal tumors (CNS-PNET) have undergone a major re-organization in the recent WHO classification, to reflect their molecular basis [90]. They encompass a range of histological subtypes including embryonal tumor with multi-layered rosettes (ETMR), CNS neuroblastoma, CNS ganglioneuroblastoma and medulloepithelioma.

Amplification of the C19MC oncogenic miRNA cluster and high LIN28 expression has been linked to an aggressive group of embryonal tumors which span histological subtypes [9]. PI3K/mTOR pathway genes were found to be enriched within this subgroup. Immunohistochemical analysis additionally revealed up-regulation of phosphorylated S6 (indicating active mTOR signaling) [9]. Pharmacological inhibition of PI3K/mTOR signaling inhibited the growth of CNS PNET cells in culture suggesting this is a potential therapeutic target [9, 91].

## THERAPEUTIC TARGETING OF PI3K SIGNALING IN PEDIATRIC BRAIN TUMORS

The high frequency of alterations in PI3K signaling in pediatric brain tumors has led to the initiation of clinical trials testing these agents (Table 1). The most advanced target downstream mTOR signaling.

**Table 1:** Current and completed clinical trials targeting PI3K signaling in pediatric CNS tumors

Drug	Target	Study type	Tumor type	Result	Reference
MK2206	AKT	Phase I	Refractory tumors	Well tolerated. Phase II dose identified	103
Perifosine	AKT	Phase I	Recurrent solid tumors		NCT00776867
Perifosine (+temsirolimus)	AKT	Phase I	Recurrent solid tumors		NCT01049841
Everolimus	mTOR		SEGA	Approved for treatment	36
Everolimus	mTOR	Phase I	Recurrent/refractory solid tumors	Well tolerated with significant inhibition of mTOR signaling. MTD identified	92
Everolimus	mTOR	Phase II	Recurrent LGG	Stable disease in 12/23 patients and partial response in 4/23	93
Everolimus	mTOR	Phase II	Progressive LGG	-	NCT01734512
Everolimus	mTOR	Phase II	Recurrent/progressive ependymoma	-	NCT02155920
Everolimus	mTOR	Feasibility	Refractory/recurrent brain tumors	-	NCT02015728
Everolimus	mTOR	Phase II	DIPG	-	NCT02233049
Temsirolimus	mTOR	Phase I	Recurrent/refractory solid tumors	Well tolerated. Anti-tumor activity and inhibition of mTOR signalling. MTD not identified	94
Temsirolimus	mTOR	Phase II	HGG, neuroblastoma, rahbdomyosarcoma	Did not meet objective efficacy threshold for continuation, but suggested further study.	95
Temsirolimus (+irinotecan, temozolomide)	mTOR	Phase I	Relapsed/refractory solid tumors	Well tolerated. MTD identified.	96
Temsirolimus (+cixutumumab)	mTOR	Phase I	Recurrent solid tumors	Well tolerated. Phase II dose identified.	97
Temsirolimus (+cixutumumab)	mTOR	Phase I	Recurrent/refractory solid tumors		NCT00880282
Temsirolimus (=bevacizumab, cyclophosphamide, valproic acid)	mTOR	Phase 0	Solid tumors at high risk of recurrence		NCT02446431
Rapamycin (+erlotinib)	mTOR	Feasibility/ Phase II	Recurrent LGG	Well tolerated. Questionable to no activity.	98
Rapamycin (+vinblastine)	mTOR	Phase I	Recurrent/refractory solid tumors	Well tolerated. Anti-tumor effects seen.	99
Rapamycin (+chemotherapy)	mTOR	Phase I	Recurrent/refractory solid tumors		NCT01331135
Rapamycin (+irinotecan)	mTOR	Phase I	Refractory solid tumors		NCT01282697
Rapamycin (+cyclophosphamide, topotecan)	mTOR	Phase I	Recurrent/refractory solid tumors		NCT01670175
Ridaforolimus	mTOR	Phase I	Refractory solid tumors	Well tolerated with no dose limiting toxicities identified. MTD not identified.	100
Ridaforolimus	mTOR	Phase I	Advanced solid tumors	We tolerated. MTD not identified. Phase II dose determined.	101
Ridaforolimus (+dalotuzumab)	mTOR	Phase I	Advanced solid tumors	Demonstrated reasonable tolerability	102
Everolimus and AZD2014	mTOR	Phase I/II	Recurrent/refractory tumors		NCT02813135

SEGA = subependymal giant cell astrocytoma, MTD = maximum tolerated dose, LGG = low grade glioma, HGG = high grade glioma, DIPG = diffuse intrinsic pontine glioma

Everolimus has recently been approved for treatment of TSC patients with SEGA [36]. Everolimus was also well tolerated when tested in a phase I trial of children with recurrent/refractory solid tumors and was shown to significantly inhibit mTOR signaling at the maximum tolerated dose (MTD) [92]. A recent phase II trial for children with recurrent LGG demonstrated stable disease in 13/23 patients and a partial response in four [93]. Current trials are continuing to investigate the drug in pediatric brain tumors including phase II trials investigating progressive LGG (NCT01734512) and recurrent or progressive ependymoma (NCT02155920). Additionally, two trials are investigating treatment with everolimus following selection based on the presence of relevant abnormalities in patients with refractory or recurrent brain tumors (NCT02015728) or DIPG (NCT02233049/BIOMEDE).

Temsirolimus was the first mTOR inhibitor approved for treatment in oncology. In phase I trials of pediatric patients with recurrent/refractory solid tumors temsirolimus was shown to be well tolerated [94]. In a phase II trial of children with HGG, neuroblastoma and rhabdomyosarcoma, temsirolimus treatment did not meet the objective efficacy threshold for continuation [95]. However, prolonged disease stabilization was observed and supported further study. A phase I trial of temsirolimus in combination with irinotecan and temozolomide in children, adolescents and young adults with relapsed/refractory solid tumors found the combination well tolerated [96]. Similarly a phase I study of children with recurrent solid tumors demonstrated the combination of temsirolimus with cixutumumab, a monoclonal antibody targeted against the insulin-like growth factor-1 receptor (IGF-1R), to be well tolerated [97]. A second phase I study investigating the combination of temsirolimus with cixutumumab in younger patients with solid tumors which have recurred or not responded to treatment is ongoing (NCT00880282). Additionally, a phase 0 study has begun looking at the potential for use of metronomic therapy, defined as lower dose continuous drug exposure, which includes temsirolimus, in patients with solid tumors at high risk of recurrence (NCT02446431).

A study investigating the efficacy of rapamycin in combination with the EGFR inhibitor erlotinib in children with recurrent LGG was well tolerated but had questionable to no activity [98]. A second phase I trial investigating the combination of rapamycin with vinblastine, in pediatric patients with recurrent or refractory solid tumors, was found to be safe and well tolerated with preliminary evidence of anti-tumor effects [99]. Ongoing phase I trials are investigating rapamycin in combination with chemotherapy in children with recurrent/refractory solid tumors (NCT01331135, NCT01282697, NCT01670175).

A phase I study of ridaforolimus in pediatric patients with refractory solid tumors demonstrated the drug to be well tolerated [100]. In a second phase I trial of pediatric patients with advanced solid tumors ridaforolimus was well tolerated and a phase II dose was identified [101]. A further phase I trial investigating ridaforolimus in combination with the IGF-1R inhibitor dalotuzumab showed reasonable tolerability [102].

A recently opened proof of concept therapeutic stratification trial in children, adolescents and young adults with relapsed or refractory tumors aims to use molecular analysis to identify treatment targets. The trial includes use of everolimus and the dual mTORC1 and 2 inhibitor AZD2014 (NCT02813135).

Trials investigating inhibitors of upstream PI3K signaling are less advanced. The AKT inhibitor MK2206 was investigated in a phase I trial of children with refractory malignancies where the drug was well tolerated [103]. The AKT inhibitor perifosine is currently being investigated as a single agent in a phase I trial of recurrent pediatric solid tumors (NCT00776867) and in combination with temsirolimus in a second trial (NCT01049841).

Further PI3K inhibitors have been investigated pre-clinically as part of the pediatric preclinical testing program. Studies investigating the PI3K inhibitor XL146, the AKT inhibitor GSK690693 and the mTOR inhibitor MLN0128 all demonstrated modest anti-tumor activity in preclinical models [104–106].

## CONCLUSIONS

The PI3K signaling pathway has been implicated in a high proportion of pediatric CNS tumors suggesting it represents a promising target for therapy. Pathway activation has been identified in primary tumors and pre-clinical analysis has demonstrated PI3K signaling plays a role in the control of tumor growth, survival and migration.

Pre-clinical analysis of pathway inhibitors also strongly supports the potential therapeutic use of agents targeting PI3K signaling in pediatric CNS malignancies, with inhibitors able to impair the growth and migration of tumor models as well as enhancing the cytotoxic effects of chemotherapeutic agents. Initial clinical trials have been promising with agents generally well tolerated. The evidence is particularly strong for the use of mTOR inhibitors, with approval of everolimus for the treatment of SEGA patients.

It will however be important to take into consideration problems encountered in other cancers, including adult glioma, where effects of inhibitors so far have, in general, been modest [30]. This may be due in part to poor patient selection, where predictive biomarkers have not been used to identify and select patients with a responsive tumor. Secondly, as with many targeted therapies, resistance mechanisms, such as activation of compensatory signaling, are likely to develop and therefore limit effectiveness [107]. Cross talk between different signaling pathways is complex with alternative mechanisms able to control the same downstream targets. Knowledge of how alternative signaling mechanisms are involved in a specific cancer is important when planning how to target it therapeutically. One example of this is the MAPK signaling pathway which can also activate mTOR signaling [13]. MAPK signaling is commonly activated in pediatric low grade gliomas through BRAF fusions suggesting this rather than PI3K signaling may commonly activate mTOR signaling in these tumors [108, 109]. It will also be important to investigate effects of inhibitors on tumor stroma and how this might impact clinical use of PI3K pathway inhibitors [40].

A further issue specific to brain tumors is the ability of a drug to cross the blood brain barrier and achieve high enough concentrations within the brain without systemic toxicity. Caution must also be undertaken when targeting key cellular signaling pathways that are fundamental to normal development in childhood. Evaluation of toxicity to the normal brain therefore needs to be taken into consideration.

To overcome these challenges it will firstly be important to develop sophisticated patient selection strategies, using predictive biomarkers, to ensure patients entered into a trial have a responsive tumor. Secondly, a combination approach will be needed using PI3K inhibitors alongside conventional treatments as well as other novel targeted agents to increase effectiveness and avoid development of resistance. The best and most rational combinations will need to be identified. This can only be achieved if we increase our understanding of the underlying biology of these tumors and continue analysis using pre-clinical models.
